# Polythioethers bearing side groups for efficient degradation by E1cB reaction: reaction design for polymerization and main-chain scission†

**DOI:** 10.1039/d3ra03751g

**Published:** 2023-07-10

**Authors:** Ryo Kawatani, Keito Hagiwara, Anri Tanaka, Yasuhiro Kohsaka

**Affiliations:** a Faculty of Textile Science and Technology, Shinshu University 3-15-1 Tokida Ueda Nagano 386-8567 Japan kohsaka@shinshu-u.ac.jp; b Research Initiative for Supra-Materials (RISM), Interdisciplinary Cluster for Cutting Edge Research (ICCER), Shinshu University 4-17-1 Wakasato Nagano City Nagano 380-8553 Japan

## Abstract

We have previously reported the polycondensation by the tandem reactions of dithiols and α-(bromomethyl)acrylates, consisting of conjugate substitution (S_N_2′ reaction) and conjugate addition (Michael addition) reactions. The resulting polythioethers underwent a main-chain scission (MCS) by E1cB reaction, which is the reverse reaction of conjugate addition, although it was not quantitative due to the equilibrium. Herein, the modification of the structures of polythioethers led to irreversible MCS, whereby the β-positions of ester moieties were substituted with a phenyl group. This slight modification in the polymer structure influenced the monomer structures and polymerization mechanisms. The understanding of reaction mechanisms by model reactions was required to obtain high molecular weights of polythioethers. It was clarified that the consequent additions of 1,4-diazabicyclo[2.2.2]octane (DABCO), 1,8-diazabicyclo[5.4.0]undec-7-ene (DBU), and PBu_3_ were effective to achieve high molecular weight. The resulting polythioethers decomposed by irreversible MCS *via* E1cB reaction with DBU.

## Abstract 2

A pendant group led to the irreversible main chain scission of polythioether by E1cB reaction. The pendant group also led to divergent polymerization paths according to catalysts, and the optimization by understanding the mechanism was required.

## Graphical abstract

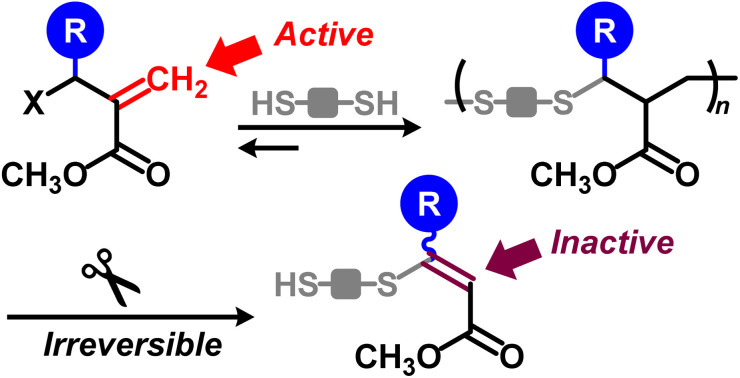

## Introduction

The main-chain scission (MCS) of polymers leads to a high decrease in molecular weight through a small number of reactions. As a result, the thermal properties, such as glass transition temperature (*T*_g_) and melting temperature (*T*_m_), mechanical properties, such as Young modulus and elasticity, and the solubility were drastically changed. The changes in physical properties by MCS have been applied to photoresists,^1^ dismantling adhesions,^2^ degradable crosslinked polymers,^3^ and a prospective strategy for controlling and supporting biodegradation.^4–6^ Therefore, the developments of polymers accepting MCS by specific stimuli are important issues.

Although the method to break a carbon–carbon (C–C) covalent bond is limited,^7–11^ those of carbon–heteroatom (C–X) are often performed.^12–16^ Recently, Hoye *et al.* applied the E1cB reaction (retro-oxa-Michael addition) to the MCS of polyester.^17^ The polyester was prepared by ring-opening polymerization of a δ-lactone derivative bearing a carbonyl pendant at the γ-position, and the E1cB reaction by 1,8-diazabicyclo[5.4.0]undec-7-ene (DBU) resulted in MCS producing an acrylate monomer at a high yield (95%). This study suggests that the E1cB reaction is effective for quantitative and selective MCS.

Polythioethers are typically synthesized by the polyaddition of thiols with alkenes^18^ or alkynes,^19^ whereas the radical ring-opening polymerization of cyclic α-(thiomethyl)acrylates is another practical route.^20^ The former, polyaddition, are based on thiol–ene and thiol–yne click reaction that progresses quantitatively even under ambient condition. Thiol–ene reactions include both radical and ionic mechanisms.^21^ The latter is the so-called Michael addition reaction between thiols and α,β-unsaturated carbonyl compounds, such as acrylates, which is reverse of the E1cB reaction. Therefore, optimization of molecular and reaction designs would lead to both polymerizations by Michael addition and efficient MCS by E1cB reaction under suitable conditions.

**Scheme 1:**
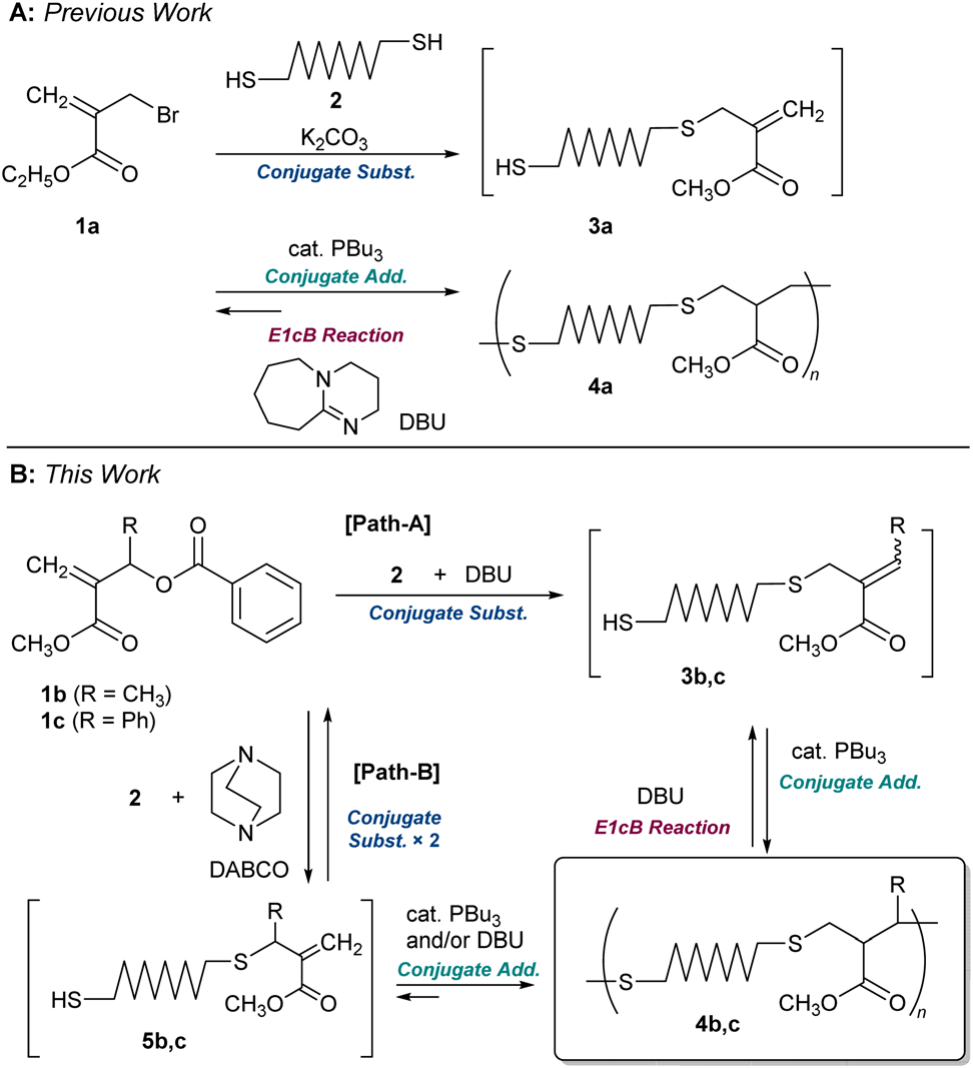
Synthesis and MCS of polythioethers. (A) Our previous report.^22^ (B) This report.

We recently reported the polycondensation *via* a tandem reaction of conjugate substitution and addition reactions of α-(bromomethyl)acrylate 1a and dithiol 2 (Scheme 1A).^16,22^ The product, 4a, underwent MCS *via* E1cB reaction in the presence of DBU.^15^ However, since the E1cB reactions were not irreversible, the end-capping of formed thiol-end with monothiol was required to promote MCS. However, the MCS was not quantitative; for example, 4a (*M*_n_ = 10 700 and *Đ* = 1.89) decomposed to an oligomer (*M*_n_ = 2100 and *Đ* = 1.83) even in the presence of 5.0 equimolar monothiol.

Herein, new polythioethers 4b and 4c, bearing methyl and phenyl substituents next to the sulphur atom, respectively, were designed to achieve more efficient MCS (Scheme 1B). The allylic substituents were found as the key leading to the MCS by the irreversible E1cB reaction. On the other hand, substitution at the allylic position complicated the polymerization. An understanding of the reaction mechanism and careful examination of the conditions were required to achieve a high degree of polymerization.

## Results and discussion

### Molecular design and model reactions

For the difficulty in synthesis, 1b and 1c, which have no halogen atom but a benzoyloxy group as a leaving group for conjugate substitution, were prepared. Two routes are possible to access 4b:^23^ one is a direct conjugate substitution with 2, and the subsequent conjugate addition (Scheme 1B, path A). As this is a reversible reaction, the reaction condition is expected to be the key to achieving a high degree of polymerization. The other is the conjugate addition reaction of 5b, which seems more reactive than 3b due to the *exo*-olefin structure (path B). Thus, we initially planned the polymerization *via* path B. For this strategy, the issue of how 5b can be prepared from 1b must be addressed.

**Scheme 2:**
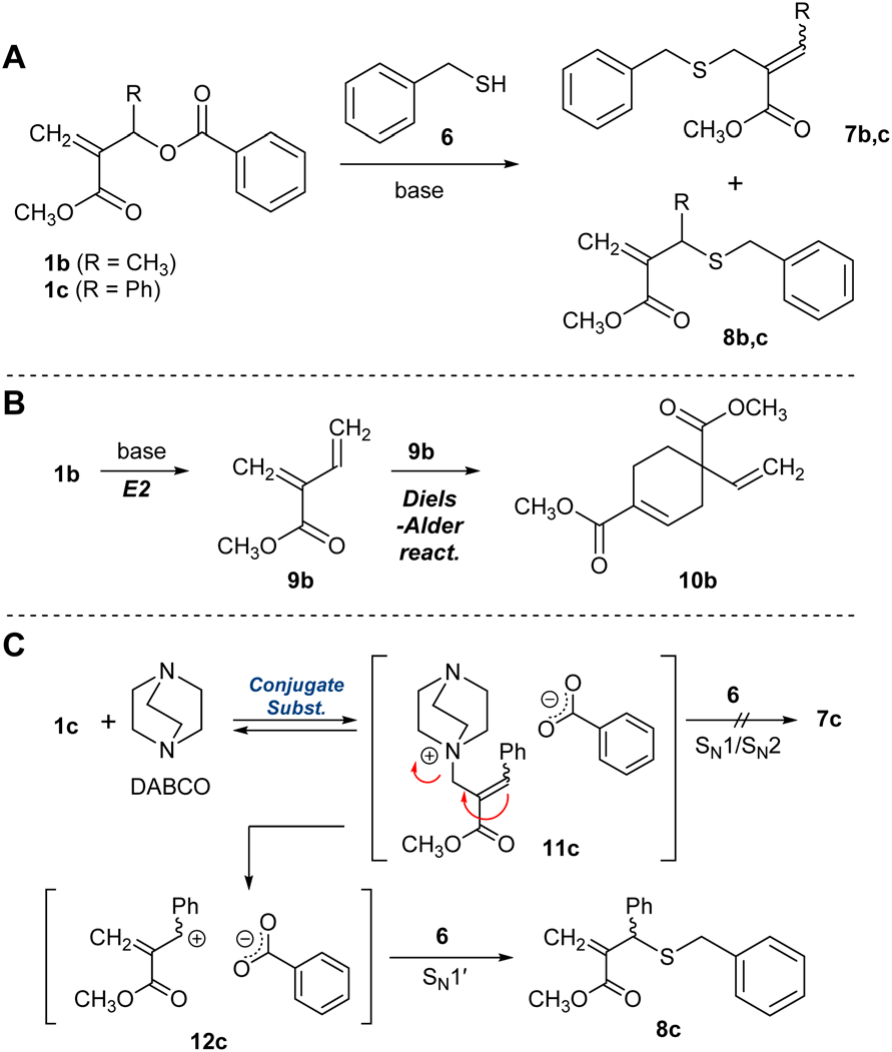
A model reaction of 1b and c with 6 (A) and the proposed reaction mechanisms (B) and (C).

Yu *et al.* have reported the synthesis of an *exo*-methylene product by the nucleophilic substitution reaction of the analogue of 1b and a phenol catalysed by Et_3_N,^24^ which was a hint to prepare 5b from 1b, although the product yield was not quantitative. As is well known, quantitative and selective conversion is necessary for polycondensation. Therefore, a model experiment with benzyl mercaptan (6) was conducted in CDCl_3_ to optimize the reaction condition for the selective and quantitative synthesis of an *exo*-olefin 8b (Scheme 2A). Without a base, no reaction occurred (Table 1, entry 1). Then, 6 was added to a mixture of DABCO and 1b, but an E2 reaction and the subsequent Diels–Alder reaction to yield 9b and 10b, respectively, were observed (Scheme 2B, entry 2, Fig. S3†). To avoid these side reactions, 1b and 6 were mixed in advance, and DABCO was added (entry 3, Fig. S4†). The main product was *exo*-olefin 8b, with some slight side reactions. To reduce the side reactions, the feed of DABCO was decreased to a catalytic amount, but selectivity was unchanged, thus slowing down the process (entry 4, Fig. S5†). Weak bases, such as Et_3_N (entry 5) and iPr_2_NEt (entry 6), resulted in low conversion and selectivity, while a stronger base, DBU, yielded 7b as the main product (entry 7). The side reactions are not desirable for polycondensation as they lead to a low degree of polymerization. Thus, 1c, which has a phenyl group instead of methyl group at the allylic substituent, was prepared as a substrate free from the possibility of an E2 reaction.^24^ As expected, the selective formation of 8c was achieved with DABCO (entry 10, Fig. S6†). Thus, the combination of 1c and DABCO seemed suitable for path B in Scheme 1B. Notably, bases with low nucleophilicity, such as iPr_2_NEt (entry 12) and DBU (entry 13), were effective in preparing 7c. Since iPrNEt_2_ resulted in decreased conversion, a combination of 1c and DBU seemed suitable for the preparation of 7c and path A in Scheme 1B.

**Table 1:** Model experiments with benzyl mercaptan (6) using various bases

Entry^a^	1	Base^b^ (equimol.)	Time [h]	Conv.^c^ [%]	Composition^c^ [%]			
					7	8	9	10
1	1b	—	1	0				
2^d^	1b	DABCO (1.2)	1	86			85	15
3	1b	DABCO (1.2)	1	>99	1	95	1	2
4	1b	DABCO (0.33)	15	81	1	94	3	2
5	1b	Et_3_N (1.2)	24	26	58	38	0	4
6	1b	iPr_2_NEt (1.2)	24	Trace				
7	1b	DBU (1.2)	3	>99	96	2	1	1
8	1b	DBU (0.33)	36	27	95	1	0	4
9	1c	—	1	0				
10	1c	DABCO (1.2)	1	97	<1	>99	0	0
11	1c	Et_3_N (1.2)	24	35	61	31	0	0
12	1c	iPr_2_NEt (1.2)	24	18	>99	<1	0	0
13	1c	DBU (1.2)	1	97	97	3	0	0

a 1: 60 μmol, [1]/[6] = 1/1.2, CDCl_3_: 0.70 mL, 25 °C. A base was added after mixing 1 and 6.

b Equimolar to 1.

c Determined by ^1^H NMR spectra.

d 1b and DABCO were mixed before adding 6.

The experiments suggested the following reaction mechanism. A poor nucleophilic base, *e.g.* DBU, led to the deprotonation of 6 and the subsequent conjugate substitution reaction to yield 7c. In contrast, a nucleophilic base, such as DABCO, preferred the conjugate substitution to 1c than the deprotonation of 6 (Scheme 2C). Then, the *endo*-olefin intermediate, 11c was formed. Since the product 8c involved an S_N_1′ mechanism, *i.e.*, the elimination–substitution mechanism, was considered reasonable rather than S_N_1 and S_N_2 reaction^25^ toward 7c. In this context, the phenyl substituent might have a decisive effect on promoting the formation of 8c, as the resonance effect stabilizes the intermediate 12c.

### Polymerization

Since DBU was expected to yield the intermediate 3c selectively and quantitatively (Scheme 1B), the polycondensations of 1c and 2 through path A were conducted in the presence of DBU (Table 2, entries 1–3). Polymerization conditions were referred from our previous papers^22^ to compare polymerization behaviors with 4a (*M*_n_ = 10 700 and *Đ* = 1.89). However, lower molecular weight polymers were obtained (Table 2, entry 1, *M*_n_ = 5600 and *Đ* = 2.10). The polymerization was further monitored by size-exclusion chromatograms (SECs, Fig. S9†). The molecular weight increased after 7 h but decreased after 24 h, suggesting MCS by E1cB reaction after the propagating reaction. Therefore, the reaction system seemed to have reached equilibrium. The addition of PBu_3_ to promote the conjugate addition (propagating reaction) resulted in a slight increase in molecular weight (entries 4 and 5, Fig. S10†).

**Table 2:** Polycondensation of 1c and 2 using DBU and PBu_3_

Entry^a^	Base (equimol.)	Solvent	Temp. [°C]	Time [h]	Yield [%]	*M*_n_ b	*Đ* b
1	1.2	CHCl_3_	25	24	>99	5600	2.10
2	1.2	CH_3_CN	25	24	>99	4900	2.32
3	1.2	CH_3_CN	50	24	>99	4700	1.91
4	1.2	CH_3_CN	25	24 + 5^c^	37	6600	1.60
5	2.2	CH_3_CN	25	24 + 5^c^	28	7300	1.74

a 1c: 0.750 mmol, [1c]/[2]/[base]/[catalyst] = 1/1.0/1.2/0.2. Base: DBU, catalyst: PBu_3_, solvent: 0.75 mL.

b Determined by SEC (THF, 40 °C, polystyrene standards).

c Polycondensation for 24 h with DBU, and then, PBu_3_ was added.

**Scheme 3:**
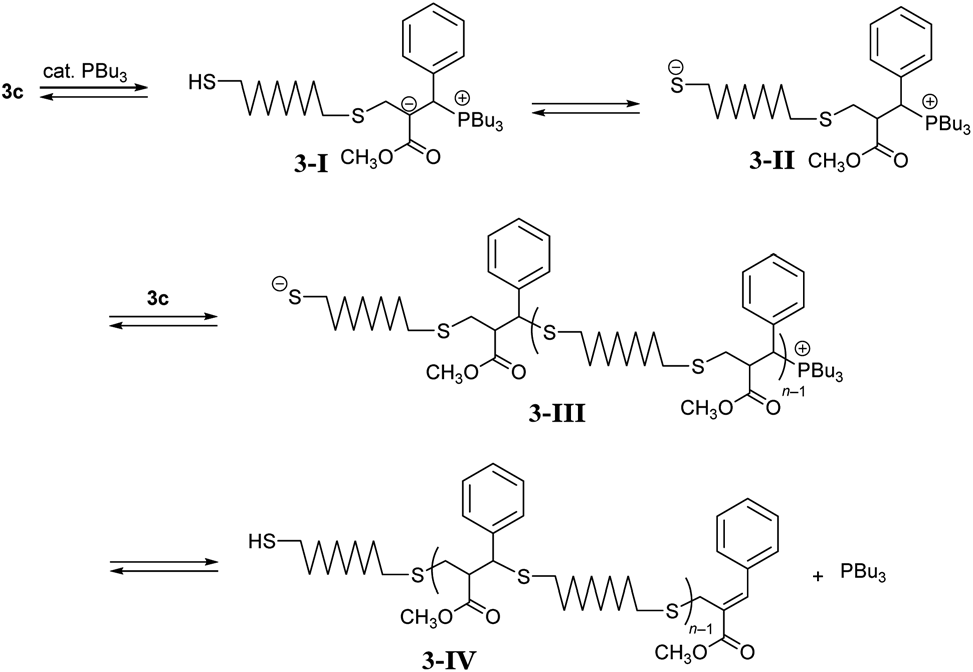
A proposed side reaction in the polycondensation of 3c catalyzed by PBu_3_.

Next, polymerizations through path B were investigated using DABCO and PBu_3_ in CHCl_3_ (Table 3, entry 1). However, the resulting product was a polymer with a low degree of polymerization (*M*_n_ = 1000 and *Đ* = 2.25). Similar results were obtained in CH_3_CN (entries 2 and 3). To investigate the reason of unsuccessful polycondensation, the reaction was monitored in CD_3_CN by ^1^H NMR spectra (Fig. S11†). After 1 h, 5c was observed as the main product, indicating reaction proceeding through path B as expected. However, signal X assigned to the *endo*-olefin proton was observed around 7.7 ppm, which became more pronounced after 16 h, suggesting an unexpected reaction that inhibited further propagation. A possible mechanism of the side reaction is described in Scheme 3. The conjugate addition proceeds through the addition of PBu_3_ to an acrylate skeleton to form enolate intermediate 3-I.^21^ The subsequent proton transfer forms thiolate anion 3-II, and the conjugate addition follows. Herein, the elimination from the phosphonium end of 3-III to the chain end 3-IV is possible. A basic catalyst, such as Et_3_N, that directly deprotonates thiols was also effective in promoting conjugate substitution,^25^ and the weak base was expected to decrease the side reaction. Thus, Et_3_N (entry 4) and iPr_2_NEt (entry 5) were analysed but found ineffective in increasing the molecular weight, probably due to the low activity. A stronger base, DBU, was more effective (entry 6, *M*_n_ = 5400, and *Đ* = 2.16), although the molecular weight was still lower than polymers obtained in entry 5 in Table 2. Stronger bases than DBU were expected to be ineffective in increasing the molecular weight because they promoted to MCS by E1cB reaction. So in the place of such stronger bases, PBu_3_, a catalyst which promote the Michael addition, was used with DBU to enhance the propagation. However, the polymerization, initiated with DABCO and promoted by adding DBU and PBu_3_ simultaneously, resulted in a similar molecular weight (entry 7). In entry 8, the reaction of 1c and 2, initiated with DABCO, was monitored by SEC (Fig. S12†). After 1 h, DBU was added to promote conjugate addition. As a result, *M*_n_ increased from 510 to 7100 after a further 1 h. Then, PBu_3_ was added and the reaction was allowed to proceed for 22 h at which point the *M*_n_ had increased to 13 000. Thus, the addition of PBu_3_ at the early stage of reaction was ineffective because of the side reaction of elimination from the phosphonium intermediate 3-III. However, the addition of PBu_3_ after the almost complete consumption of the acrylate chain end promoted further propagation. In this stage, the elimination from the chain end to prevent further propagation was not a fatal problem.

**Table 3:** Polycondensation of 1c and 2 using DABCO and catalysts

Entry^a^	Catalyst	Solvent	Temp. [°C]	Yield [%]	*M*_n_ b	*Đ* b
1	PBu_3_	CHCl_3_	25	10	1000	2.25
2	PBu_3_	CH_3_CN	25	27	1600	2.09
3	PBu_3_	CH_3_CN	50	66	1100	2.02
4	Et_3_N^c^	CH_3_CN	25	51	1900	1.78
5	iPr_2_NEt^c^	CH_3_CN	25	49	1800	2.31
6	DBU^c^	CH_3_CN	25	83	5400	2.16
7^d^	DBU + PBu_3_	CH_3_CN	25	29	5600	2.05
8^e^	DBU/PBu_3_	CH_3_CN	25	57	13 000	1.63

a 1c: 0.750 mmol, [1c]/[2]/[base]/[catalyst] = 1/1.0/1.2/0.2. Solvent: 0.75 mL.

b Determined by SEC (THF, 40 °C, polystyrene standards).

c 1c: 0.500 mmol, [1c]/[2]/[base]/[catalyst] = 1/1.0/1.2/0.2. Solvent: 0.50 mL. Catalysts were added after 1 h and the reaction was conducted for more than 22 h.

d 1c: 0.500 mmol, [1c]/[2]/[base]/[catalyst] = 1/1.0/1.2/0.2. Solvent: 0.50 mL DBU and PBu_3_ were added after 1 h, at the same time, and the reaction was conducted for more than 22 h.

e 1c: 0.500 mmol, [1c]/[2]/[base]/[catalyst] = 1/1.0/1.2/0.2. Solvent: 0.50 mL DBU and PBu_3_ were added after 1 h and 2 h, respectively, and the reaction was conducted for more than 22 h.

### Main-chain scission

**Fig. 1:**
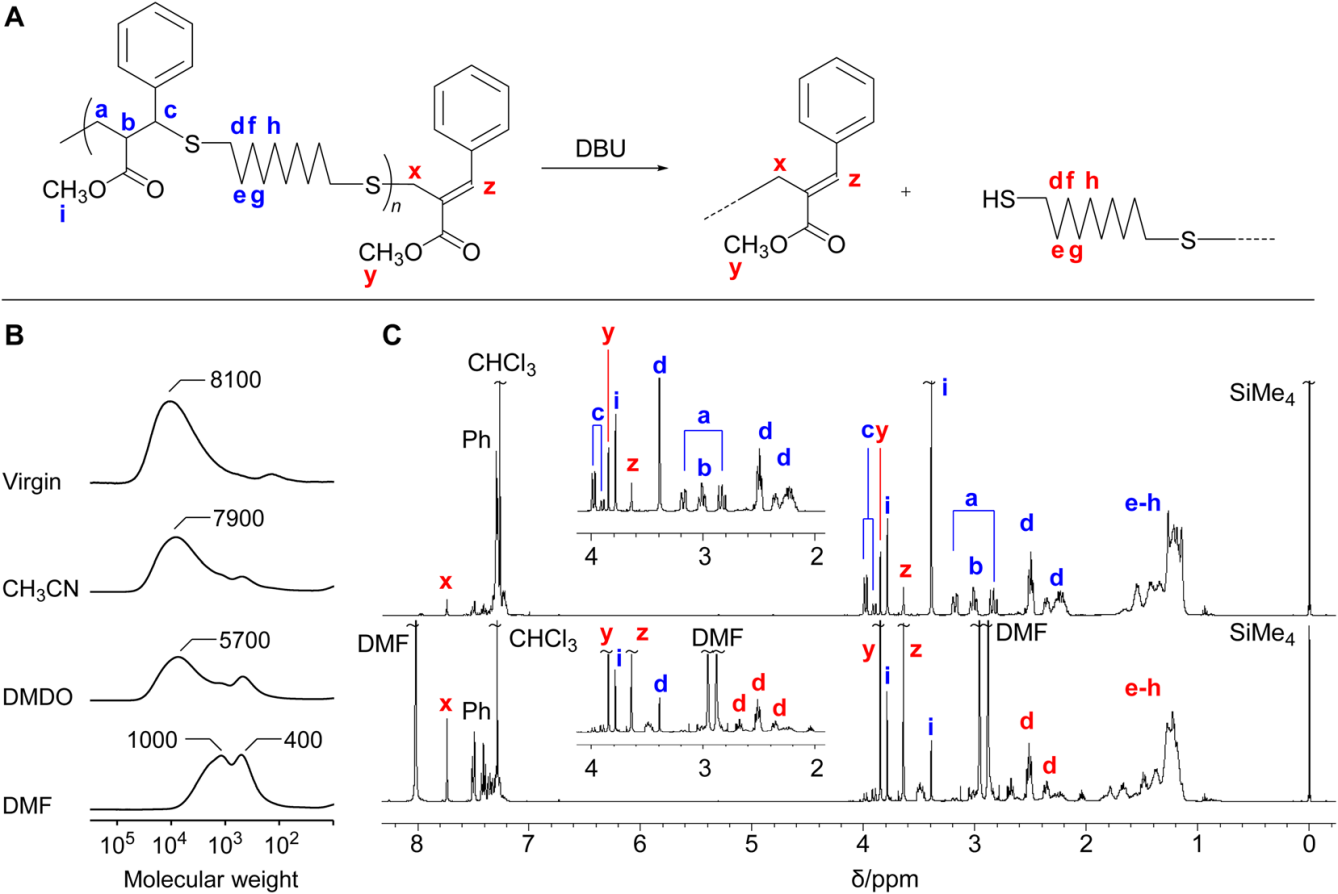
(A) MCS of 4c by E1cB reaction. (B) SECs before and after MCS in various solvents. The labels associated with peaks indicate the peak-top molecular weight (*M*_p_). (C) ^1^H NMR spectra of 4c (entry 6) and after MCS in DMF.

The polythioether 4c obtained in Table 3 (entry 6) was treated with DBU (1.2 equimolar to the repeating unit) in various solvents for 17 h (Fig. 1A). It was noted that the isolated 4c used in the MCS experiments were not completely soluble in these solvents even though the polymerization reactions in CH_3_CN reported above proceeded in a homogeneous system. As the polymer was not completely dissolved in CH_3_CN and DMSO, the MCS in these solvents resulted in incomplete degradation (Fig. 1B). On the other hand, the reaction in DMF proceeded in a homogeneous system, leading to efficient MCS to small molecules. Fig. 1C shows the ^1^H NMR spectra before and after the reaction. The signals k–m assigned to the main-chain structure were scarcely observed after the reaction, while the signals x–z specific to the chain-end structure were observed at a high intensity. These changes suggest the MCS by E1cB reaction. In this reaction, not only the change from *exo*-olefin to *endo*-olefin but also the extension of a conjugated system to cover the cinnamate-like moieties is the driving forces to shift the equilibrium from polymerization to MCS. Furthermore, the amount of effective DBU in the polymerization system should be lower than that of MCS experiment, even if equal amount of DBU was used; in polymerization system, the acetic acid was formed as a byproduct in the conjugate substitution reaction, which played to deactivate the DBU. In addition, PBu_3_ contributed equilibrium shift to the propagation side. In other words, the MCS reaction of the obtained polymers, employed using higher effective concentration of DBU and in the absence of PBu3, resulted in effective degradation.

## Conclusions

In short, the designed polythioether, 4c, underwent efficient MCS by E1cB reaction. The incorporation of a phenyl substituent was significant both in the polymerization and MCS: in polymerization, the phenyl substituent led to the formation of an inactive *endo*-olefin chain-end, while it was a key to achieving efficient MCS. In conventional polymer chemistry, the modification of the backbone structure has been the typical strategy to realize MCS, and the introduction of a ‘weak’ or ‘dynamic’ covalent bond is always in discussion.^26,27^ In contrast, the above results cast a spotlight on the design of the side group. In this study, polymerization (path A) and MCS were based on the same equilibrium system. However, the polymerization through a different route (path B) enabled a high molecular weight (*M*_n_ > 10^4^). Therefore, the molecular weight increased compared to a complete equilibrium system. From the above points discussed, our conclusion is that ‘side-group design’ and ‘backbone design’ are important for both polymerization and MCS.

## Author contributions

K. H. proposed the plan and employed experiments except those for Fig. S10–S12,† which were conducted by A. T., R. K. repeated the model experiments and proposed the reaction mechanism by S_N_1′ reaction. The draft of this article was written by R. K. and Y. K. Y. K. supervised the research project.

## Conflicts of interest

There are no conflicts of interest to declare.

## Supplementary Material

RA-013-D3RA03751G-s001
