# Supplementary material for: Polythioethers bearing side groups for efficient degradation by E1cB reaction: reaction design for polymerization and main-chain scission
Source: RSC Adv. 2023 Jul 10;13(30):20782–6. doi: 10.1039/d3ra03751g (PMC10332248; doi:10.1039/d3ra03751g)
Supplement: RA-013-D3RA03751G-s001 [file RA-013-D3RA03751G-s001.pdf]

Electronic Supplementary Material (ESI) for RSC Advances.
This journal is © The Royal Society of Chemistry 2023

Polythioethers Bearing Side Groups for Efficient Degradation by E1cB Reaction: Reaction Design for Polymerization and Main-Chain Scission

Ryo Kawatani,[a] Keito Hagiwara,[a] Anri Tanaka[a] and Yasuhiro Kohsaka[a,b]*

[a] *Faculty of Textile Science and Technology, Shinshu University, 3-15-1 Tokida, Ueda, Nagano386-8567, Japan. Email: kohsaka@shinshu-u.ac.jp*

[b] *Research Initiative for Supra-Materials (RISM), Interdisciplinary Cluster for Cutting Edge Research (ICCER), Shinshu University.*

Electric Supplementary Information

Instruments

^{1}H and ^{13}C NMR spectra were recorded in $CDCl_3$ (Kanto Chemical) and CD_3CN (Kanto Chemical) on AVANCE NEO (Bruker) spectrometers. Chemical shifts in ^{1}H and ^{13}C NMR spectra were referred to the signal of tetramethylsilane (TMS) and solvent ($CDCl_3$), respectively. Molecular weight and its distributions were determined at 40 °C by size-exclusion chromatography (SEC) on an EXTREMA chromatograph (JASCO) equipped with two SEC columns [Shodex HK-404L×2], using tetrahydrofuran (THF containing 3,5-di-*tert*-butyl-4-hydroxy-toluene, Wako Pure Chemical Industries, for HPLC grade) as an eluent (flow rate = 0.6 mL min^{-1}), and calibrated against standard polystyrene (PS) samples (TSK-gel oligomer kit, Tosoh, M_n: 1.03×10^6, 3.89×10^5, 1.82×10^5, 3.68×10^4, 1.36×10^4, 5.32×10^3, 3.03×10^3, 8.73×10^2) and detected with UV (UV-4070, JASCO) and RI (RI-4035, JASCO) detectors.

Materials

Methyl acrylate, acetaldehyde, 1,4-diazabicyclo[2.2.2]octane (DABCO), 1,10-decanedithiol, benzyl mercaptan were purchased from Tokyo Chemical Industry Co., Ltd. Sodium sulfate (Na_2SO_4), pyridine, hexane, ethyl acetate (EtOAc), chloroform ($CHCl_3$), dichloromethane (CH_2Cl_2), tetrahydrofuran (THF), acetonitrile (CH_3CN), toluene, *N,N*-dimethylformamide (DMF), dimethyl sulfoxide (DMSO), 1,4-dioxane, methanol (MeOH), hydrochromic acid (HCl aq.), 1,8-diazabicyclo[5.4.0.]undec-7-ene, Et_3N, and *i*PrNEt were purchased from Fujifilm Wako Pure Chemical Industries, Ltd. Benzoyl chloride was kind gift from Iharanikkei Chemical Industry Co., Ltds.

Synthesis

Methyl 2-(1-hydroxyethyl)acrylate (13b):[1] Methyl acrylate (25.8 g, 300 mmol) and 1,8-diazabicyclo[2.2.2]octane (11.2 g, 100 mmol) were added to a solution of acetaldehyde (4.41 g, 100 mmol) in a cosolvent of 1,4-dioxane and H_2O (v/v = 1/1) at 25 °C. After 24 h, the mixture was extracted with CH_2Cl_2 (100 mL × 2). The combined organic layer was washed with brine (100 mL) and dried over Na_2SO_4. The organic layer was concentrated to yield a crude **13b** (6.06 g, yield: 46.2%). The obtained **13b** was used in the next reaction without further purification.

^{1}H NMR spectrum (400 MHz, $CDCl_3$, 25 °C): δ/ppm 6.22 (1H, s, C*H*), 5.83 (1H, s, C*H*=), 4.63 (1H, quin, *J* = 6.50 Hz, C*H*), 3.80 (3H, s, OCH_3), 2.70 (1H, d, *J* = 5.44 Hz, O*H*), 1.39 (3H, d, *J* = 6.50 Hz, CCH_3)

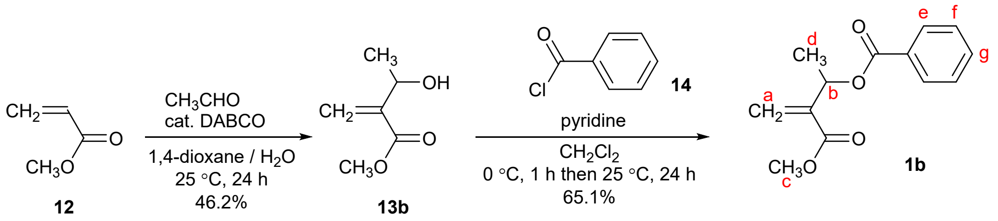

Scheme S1. Preparation of **1b**.

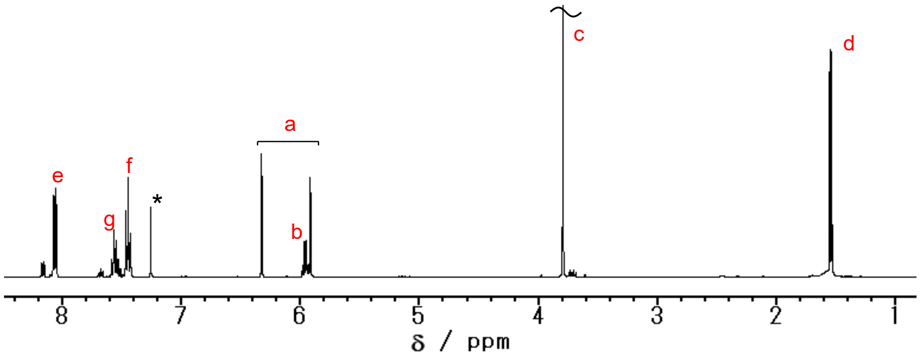

Fig. S1. ^1H NMR spectrum of **1b** (400 MHz, $CDCl_3$, 25 °C). Labels for assignments are corresponding to Scheme 1.

Methyl 2-(1-benzoyloxyethyl)acrylate (1b):[1] Pyridine (4.35 g, 55.0 mmol) was added to a solution of **1b** (6.51 g, 50.0 mmol) in CH_2Cl_2 (80 mL) at 0 °C. After 10 min, the solution of benzoyl chloride (9.14 g, 65.0 mmol) in CH_2Cl_2 (20 mL) was added dropwise to the mixture at 0 °C. After 1 h, the mixture was stirred at room temperature for 24 h. Then, H_2O (100 mL) was added at 0 °C, and the organic layer was collected. The aqueous layer was extracted with CH_2Cl_2 (50 mL × 2). The combined organic layers were washed with H_2O (100 mL) and brine (150 mL), dried over Na_2SO_4 and concentrated to yield crude **1b**. The crude product was purified by distillation under reduced pressure using a glass tube oven (Kugel Rohr, 10 Torr/170 °C, 7.62g, 65.1%).

^1H NMR spectrum (400 MHz, $CDCl_3$, 25 °C): δ/ppm 8.07 (2H, d, *J* = 7.63 Hz, C*H*), 7.59-7.53 (1H, m, C*H*), 7.45(2H, t, *J* = 7.63 Hz, C*H*), 6.33 (1H, s, C*H*=), 5.96 (1H, q, *J* = 6.53 Hz, C*H*CH_3), 5.29 (1H, s, C*H*=), 3.80 (3H, s, OCH_3), 1.55 (3H, d, *J* = 6.53 Hz, CCH_3)

Methyl 2-(hydroxy(phenylmethyl))acrylate (13c): Methyl acrylate (25.8 g, 300 mmol) and 1,8-diazabicyclo[2.2.2]octane (6.50 g, 57.9 mmol) were added to a solution of benzaldehyde (31.8 g, 300 mmol) in cosolvent of 1,4-dioxane and H_2O (v/v = 1/1, 10 mL) at 25 °C. After 24 h, H_2O (150 mL) was added, and the mixture was extracted with CH_2Cl_2 (150 mL × 2). The organic layer was washed with brine (100 mL), dried over Na_2SO_4, concentrated and washed with hexane to yield a crude **13c** (17.8 g, yield: 30.9%). The obtained **13c** was used in the next reaction without further purification.
^1H NMR spectrum (400 MHz, $CDCl_3$, 25 °C): δ/ppm 7.40-7.33 (4H, m), 7.31-7.26 (1H, m), 6.34 (1H, s, C*H*=), 5.83 (1H, t, *J* = 1.22 Hz, C*H*=), 5.57 (1H, *J* = 5.73 Hz, C*H*OH), 3.73 (3H, s, OCH_3), 2.99 (1H, d, *J* = 5.73 Hz, O*H*)

Methyl 2-(benzoyloxy(phenylmethyl))acrylate (1c): Pyridine (5.14 g, 65.0 mmol) was added to a solution of **13c** (9.61 g, 50 mmol) in CH_2Cl_2 (40 mL) at 0 °C. After 10 min, a solution of benzoyl chloride (9.14 g, 65.0 mmol) in CH_2Cl_2 (40 mL) was added dropwise to the mixture at 0 °C. After 1 h, the mixture was stirred at 25 °C for 24 h. Then, H_2O (150 mL) was added to the reaction mixture at 0 °C, and the organic layer was collected. The aqueous layer was extracted with CH_2Cl_2 (20 mL × 2). The combined organic layers were washed with 0.1

M HCl aq (150 mL × 2) and brine (150 mL). The combined organic layer was dried over Na_2SO_4 and concentrated to yield crude **3-4**. The rude product was purified on silica gel column chromatography (hexane/EtOAc = 5/1, v/v) using Wakogel C-400HG (280 g) and recrystallization in hexane (4.44g, yield: 30.0%).

^{1}H NMR spectrum (400 MHz, $CDCl_3$, 25 °C): δ/ppm 8.09-8.07 (2H, m), 7.55-7.59 (1H, m), 7.49-7.42 (4H, m), 7.38-7.29 (3H, m), 6.93 (1H, s, CC*H*), 6.45 (1H, s, C*H*=), 5.97 (1H, t, *J* = 1.07Hz, C*H*=), 3.73 (3H, s, OC*H*$_3$),

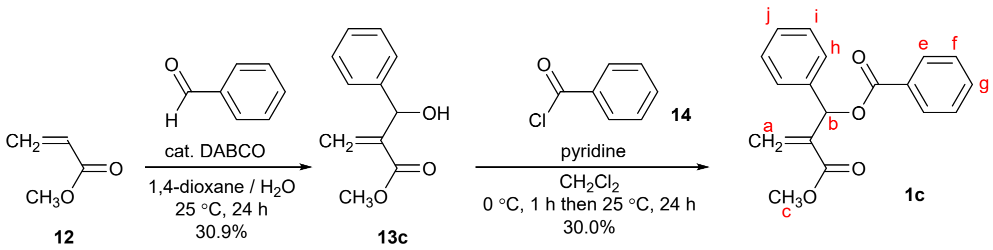

Scheme S2. Preparation of **1c**.

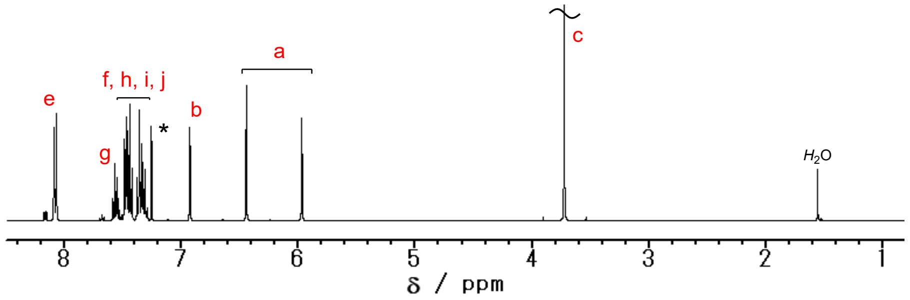

Fig. S2. ^{1}H NMR spectrum of **1c** (400 MHz, $CDCl_3$, 25 °C). Labels for assignments are corresponding to Scheme 1.

Model experiment: A typical procedure is shown (Table 1, Entry 4). A solution of benzyl mercaptan (**6**, 8.1 mg, 0.072 mmol) in $CDCl_3$ (0.5 mL) was added to a solution of **1b** (14 mg, 0.060 mmol) in $CDCl_3$ (0.2 mL). Then, DABCO (0.22 mg, 0.020 mmol) was added to the mixture at 25 °C. The reaction mixture was analysed by ^{1}H NMR spectroscopy.

Polymerization: A typical procedure is shown (Table 2, Entry 2). A solution of 1,10-decanedithiol (**2**, 0.155 g, 0.750 mmol) in CH_3CN (0.55 mL) and a solution of DABCO (0.101 g, 0.900 mmol) in CH_3CN (0.20 mL) was added to **1c** (0.222 g, 0.750 mmol). After 1 h, Bu_3P (0.0304 g, 0.150 mmol) was added to the reaction mixture. After 24 h, the reaction mixture was poured into CH_3OH (30 mL). The precipitate was collected by centrifugation and dried in vacuo to afford a polymer (7.8 mg, 27%).

Degradation of 4c: A typical procedure is shown. A solution of DBU (6.3 mg, 0.041 mmol) in DMF (1.0 mL) was added to **4c** (Entry 7, M_n = 5600, $Đ$ = 2.10, 12.6 mg, 0.0331 mmol for the repeating unit). After 17 h, small portion of reaction mixtures was sampled to monitor by ^{1}H NMR spectrometry and SEC.

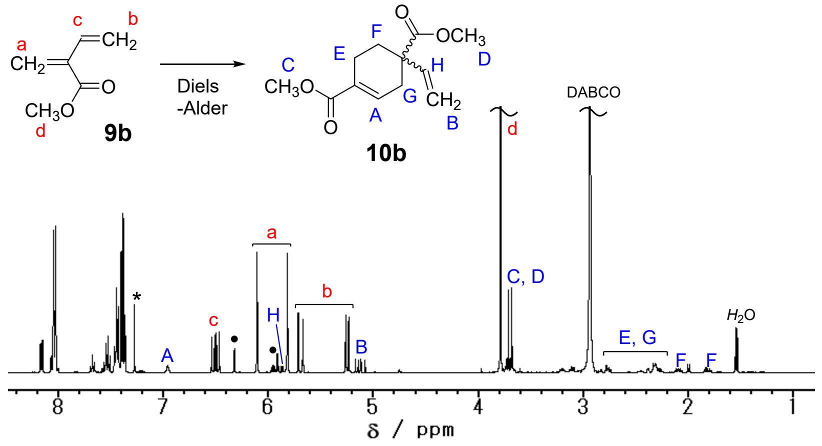

Fig. S3. ^{1}H NMR spectrum in Table 1, Entry 2. (400 MHz, $CDCl_3$, 25 °C). *: $CHCl_3$

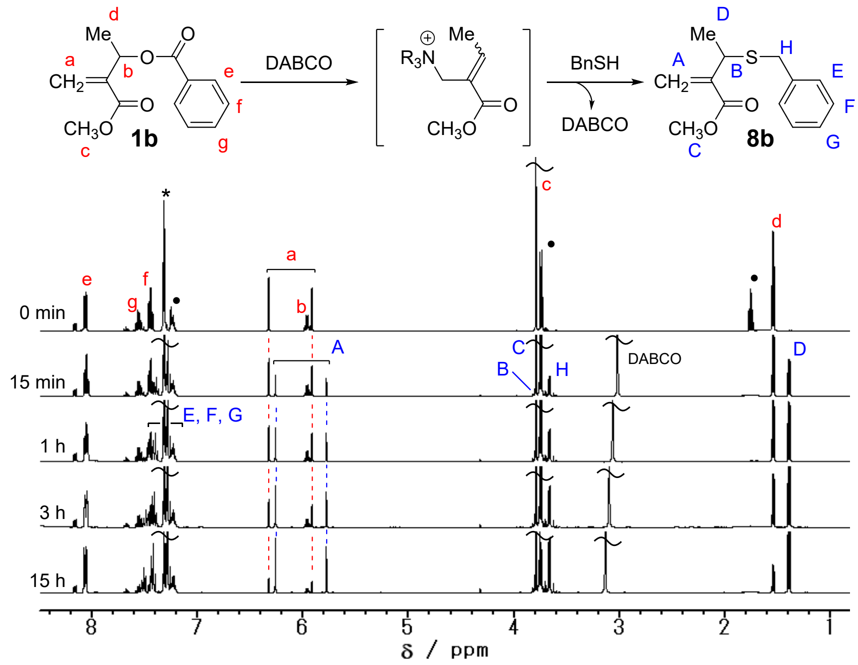

Fig. S4. ^{1}H NMR spectral changes in Table 1, Entry 3 at each time (400 MHz, $CDCl_3$, 25 °C, *: $CHCl_3$,●: **6**)

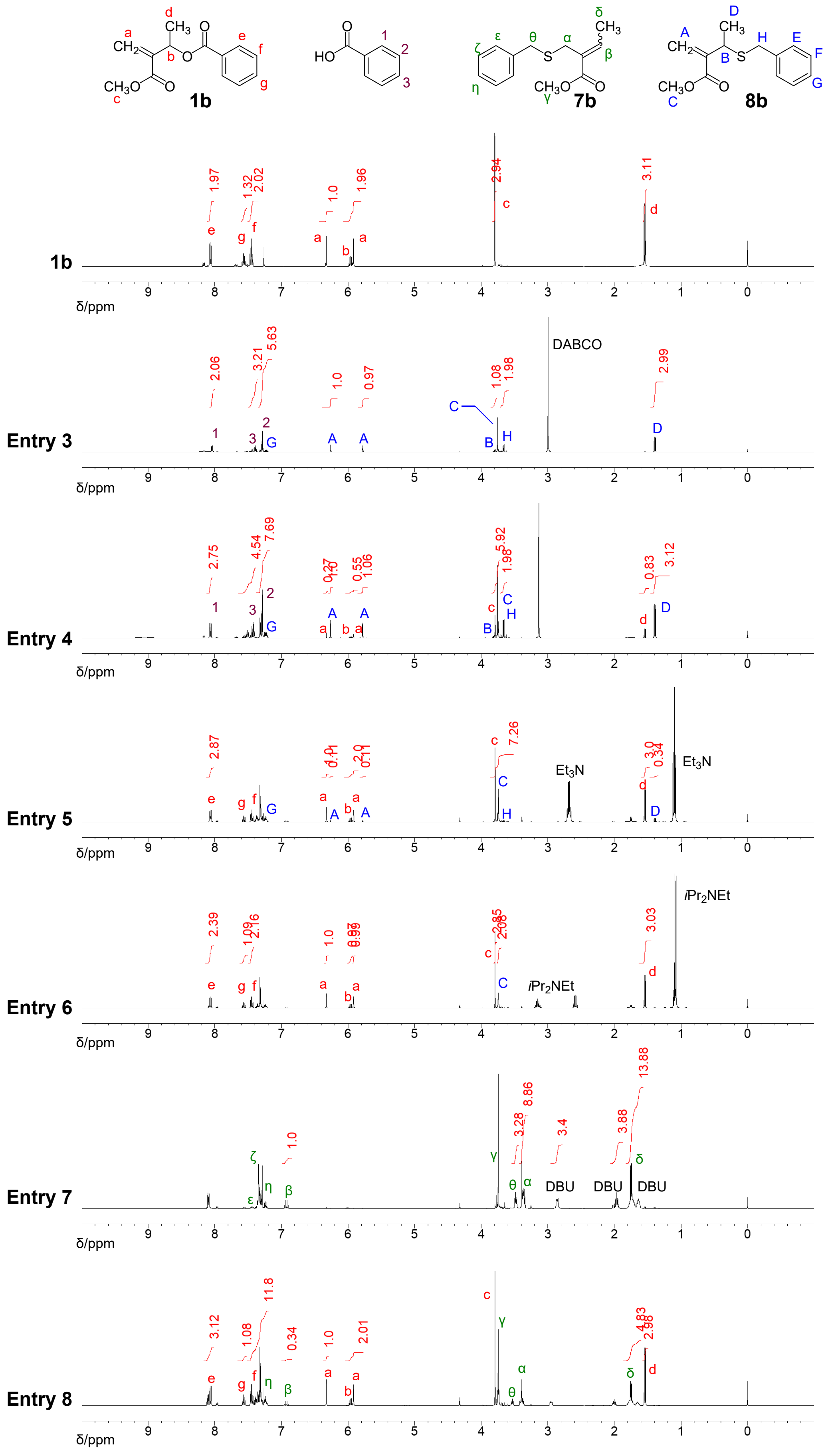

Fig. S5. ^{1}H NMR spectra of **1b** and the reaction mixture in Entries 3–8 in Table 1 (400 MHz, $CDCl_3$, 25 °C).

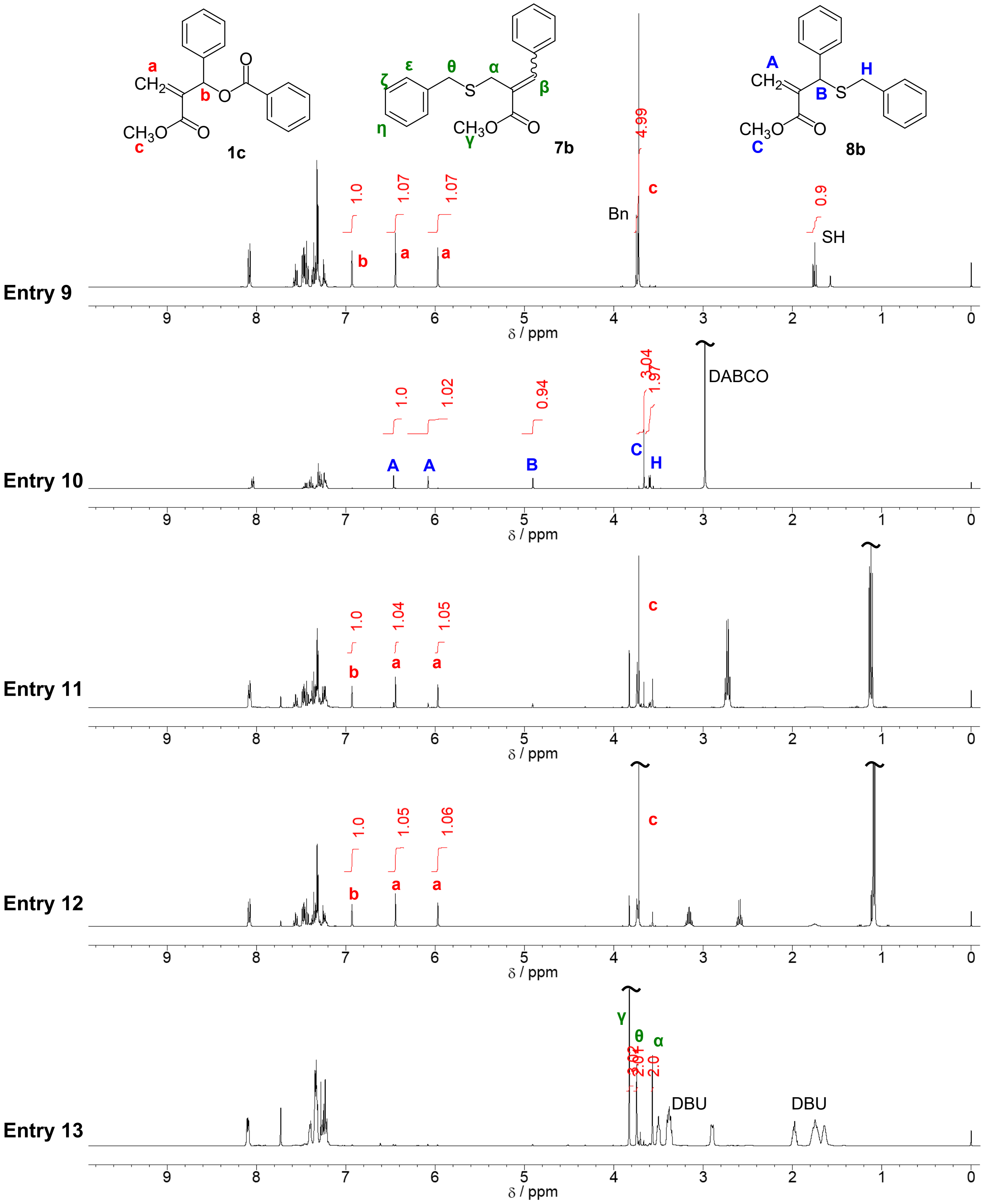

Fig. S6. ^{1}H NMR spectra of **1b** and the reaction mixture in Entries 9–13 in Table 1 (400 MHz, $CDCl_3$, 25 °C).

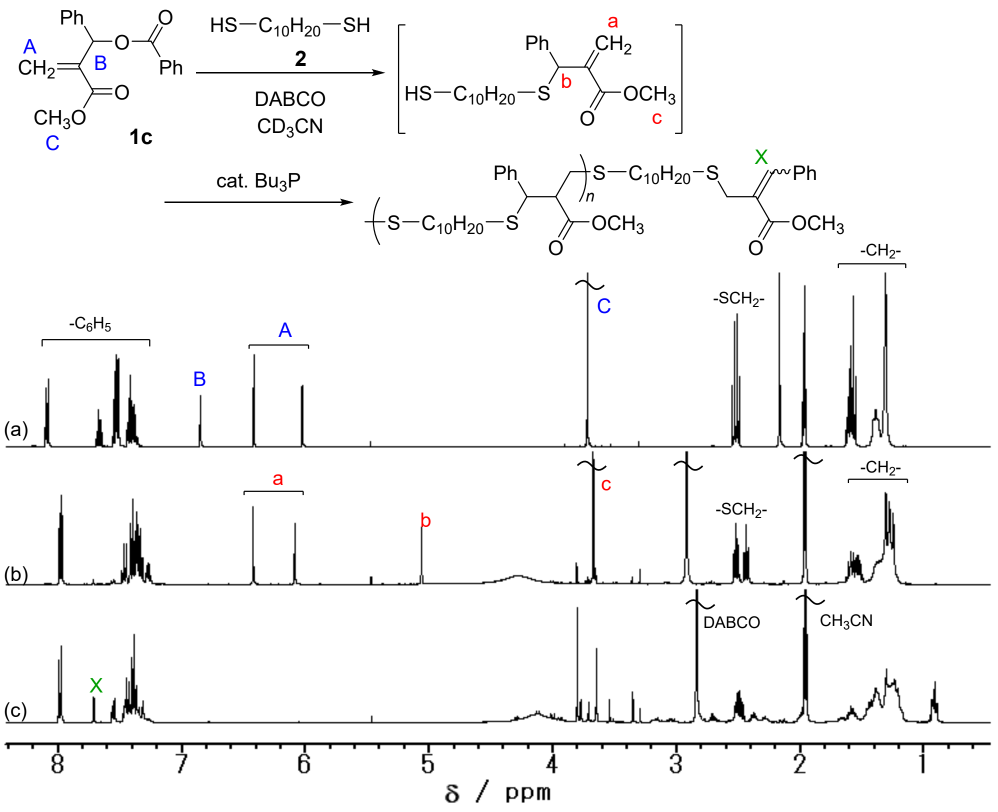

Fig. S7. ^1H NMR spectra of the reaction mixture of **1c**, **2**, DABCO and PBu_3 (a) before adding DABCO, (b) 1 h after adding DABCO and (c) 16 h after adding PBu_3. (400 MHz, CD_3CN, 25 °C).

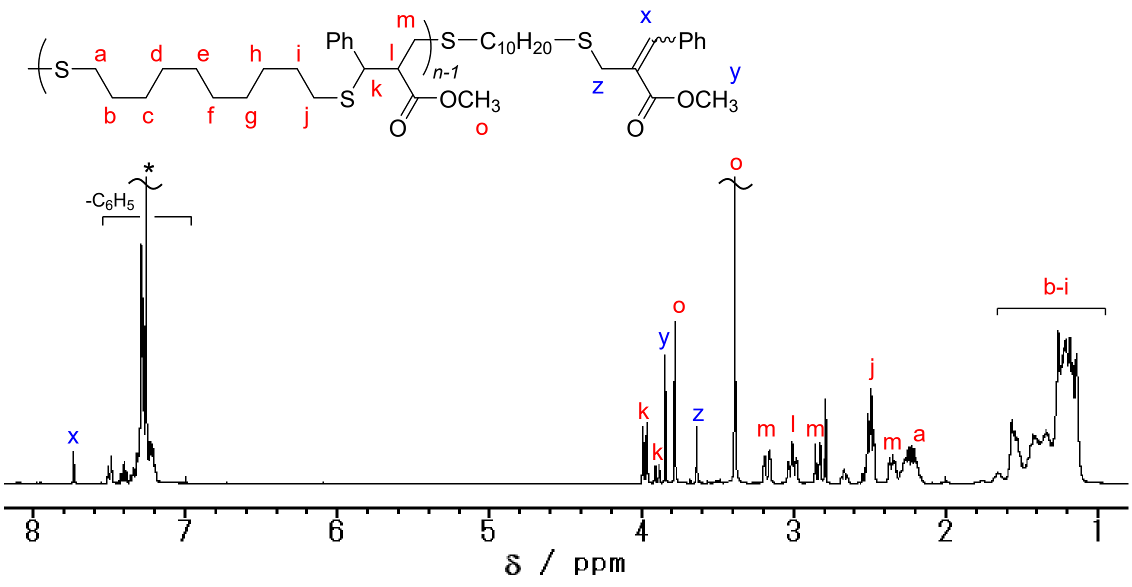

Fig. S8. ^1H NMR spectrum of the product in Entry 6 (400 MHz, $CDCl_3$, 25 °C) *:$CHCl_3$

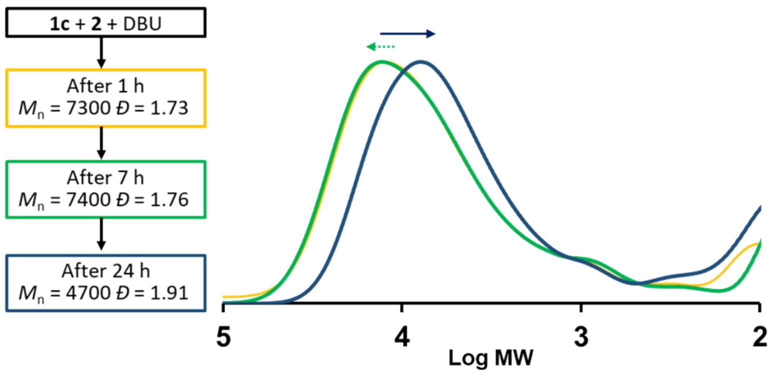

Fig. S9. Time-dependent SEC trace of polycondensation of **1c** and **2** with DBU.

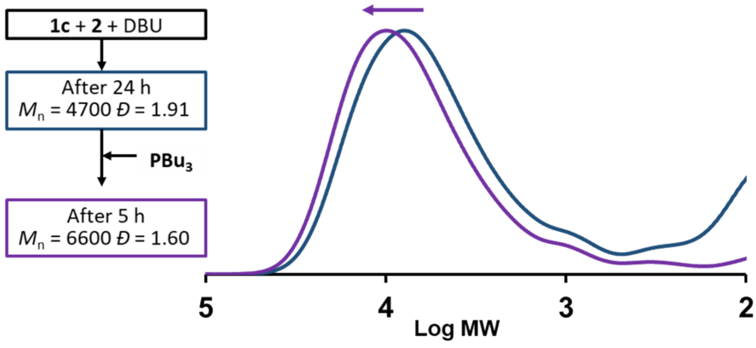

Fig. S10. Time-dependent SEC trace of polycondensation of **1c** and **2** with DBU for 24 h, and then, Bu_3P for 5 h.

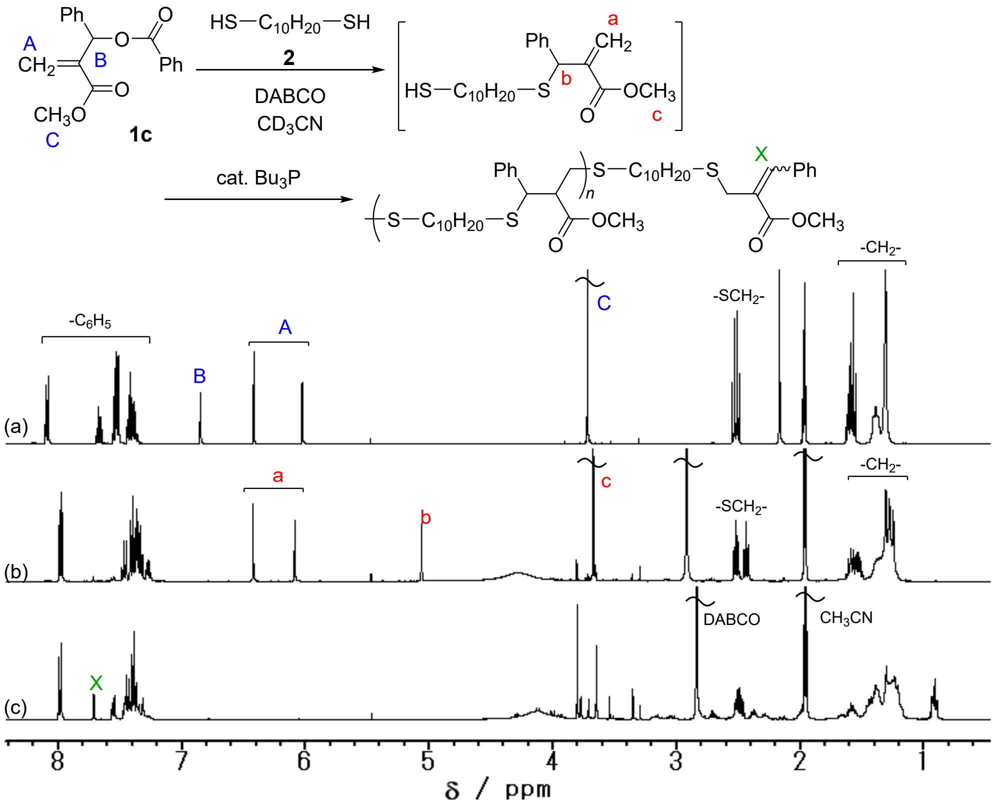

Fig. S11. ^{1}H NMR spectra of the reaction mixture of **1c**, **2**, DABCO and PBu_3 (a) before adding DABCO, (b) 1 h after adding DABCO and (c) 16 h after adding PBu_3. (400 MHz, CD_3CN, 25 °C).

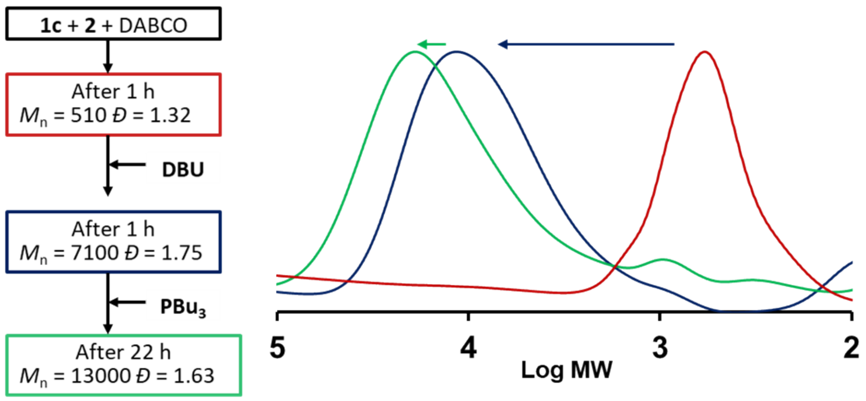

Fig. S12. Time-dependent SEC trace of polycondensation of **1c** and **2** with DABCO for 1 h, and then, DBU for 1 h, followed by PBu_3 for 22 h.

Reference

1 G. Poklukar, M. Stephan and B. Mohar, *Adv. Synth. Catal.,* 2018, **360**, 2566.
